# American Sign Language Alphabet Recognition by Extracting Feature from Hand Pose Estimation

**DOI:** 10.3390/s21175856

**Published:** 2021-08-31

**Authors:** Jungpil Shin, Akitaka Matsuoka, Md. Al Mehedi Hasan, Azmain Yakin Srizon

**Affiliations:** 1 School of Computer Science and Engineering, The University of Aizu, Aizuwakamatsu, Fukushima 965-8580, Japan; mehedi@u-aizu.ac.jp; 2 Softbrain Co., Ltd., Tokyo 103-0027, Japan; matsuoka-a@softbrain.co.jp; 3 Department of Computer Science & Engineering, Rajshahi University of Engineering & Technology, Rajshahi 6204, Bangladesh; azmainsrizon@cse.ruet.ac.bd

**Keywords:** american sign language recognition, massey dataset, finger spelling a dataset, media-pipe, distance-based features, angle-based features, support vector machine, light gradient boosting machine

## Abstract

Sign language is designed to assist the deaf and hard of hearing community to convey messages and connect with society. Sign language recognition has been an important domain of research for a long time. Previously, sensor-based approaches have obtained higher accuracy than vision-based approaches. Due to the cost-effectiveness of vision-based approaches, researchers have been conducted here also despite the accuracy drop. The purpose of this research is to recognize American sign characters using hand images obtained from a web camera. In this work, the media-pipe hands algorithm was used for estimating hand joints from RGB images of hands obtained from a web camera and two types of features were generated from the estimated coordinates of the joints obtained for classification: one is the distances between the joint points and the other one is the angles between vectors and 3D axes. The classifiers utilized to classify the characters were support vector machine (SVM) and light gradient boosting machine (GBM). Three character datasets were used for recognition: the ASL Alphabet dataset, the Massey dataset, and the finger spelling A dataset. The results obtained were 99.39% for the Massey dataset, 87.60% for the ASL Alphabet dataset, and 98.45% for Finger Spelling A dataset. The proposed design for automatic American sign language recognition is cost-effective, computationally inexpensive, does not require any special sensors or devices, and has outperformed previous studies.

## 1. Introduction

Sign language is a form of communication that utilizes visual–manual methodologies such as expressions, hand gestures, and body movements to interact among the deaf and hard of hearing community, yield opinions, and convey meaningful conversations [1]. The term deaf and hard of hearing is employed to identify a person who is either deaf or incapable to speak an oral language or have some level of speaking ability but prefer to not speak to bypass negative or undesired attention that atypical voices seldom attract.

Deafness is often expressed as hearing loss or injury which is an entire or moderate inability to hear which may appear in one or both ears of an individual [2,3]. The main reasons for hearing loss involve aging, genetics, noise exposure, a variety of infections, such as chronic ear infections, and certain toxins or medications [2]. Diagnosis of hearing loss can be practised when a person is incapable to hear 25 decibels in at least one ear after performing the poor-hearing test [2] and this test is recommended for all newborn children [4]. Hearing loss can be classified as mild (25–40 decibels), moderate (41–55 decibels), moderate-severe (56–70 decibels), severe (71–90 decibels), and profound (greater than 90 decibels) [2]. Approximately 1.33 billion people have been affected by hearing impairment to some extent, as of 2015, which covered 18.5% of the overall population of the world [5]. Similar to deafness, mutism typically denotes an absolute or moderate inability to speak. The main reasons for mutism include organic, psychological, developmental, or neurological trauma, physical disability, communication disorder, and so on [6].

Although there exist several treatments for hearing loss and mutism such as hearing aids, assistive and augmentative communication devices, sign language, cochlear implants, subtitles, etc. [2], all treatments are not commonly accepted. Statistics showed that 124 million people had moderate to severe disability as of 2013 [2,7,8] and among those, 108 million people lived in low and middle-income countries [7]. Hence, most members of deaf culture reject the efforts to cure deafness to support the community [9] and some consider the cochlear implants as concerns as they have the potential to eradicate their culture [10]. Because of these reasons sign language has become an important tool for both the deaf and hard of hearing community, and general people as a means of communication.

Previously, sign language recognition has been conducted by following two main classification mechanisms: sensor-based and vision-based recognition. Sensor-based approaches extracts the hand measurements, i.e., joints orientation, hands position, and hand velocity [11], and can be conducted using microcontrollers and specific sensors, such as data gloves [12,13], power gloves [14], digital camera [15], accelerometer [16,17], depth camera [18], Kinect [19], leap motion controller [20], dexterous master gloves [21], etc. The advantage of the sensor-based approach is the higher recognition rate because of the skeletal data [22]. However, sensor-based approaches are expensive, allow limited movement, require specialized devices, environment, and training to utilize the systems fully [22]. There is also a risk that noise will reduce the recognition rate of sensor-based systems as sensors, such as accelerometers are sensitive to noise, and even a slight movement can be identified as a waveform [22]. Hence, researchers have proposed vision-based approaches in recent years by utilizing inputs of the camera, such as web camera, stereo camera, or 3D camera [23]. These approaches are more attractive because they do not need any specialized devices with limited movement and can conduct the recognition without contact. In some cases, images with color-coded gloves have also been utilized to make hand detection easier [24]. The main advantage of vision-based approaches is that these methods are affordable and the main weaknesses are the lower recognition rate and high computing power consumption [22]. Both sensor-based and vision-based approaches can be broadly divided into two sections: feature extraction and recognition. Although the sensor-based approaches utilize different sensors and devices to acquire the skeletal data, the vision-based approaches first detect the hand and then extract desired useful features from the hand [22]. The feature extraction for sensor-based approaches is costly because of the specialized equipment. On the other hand, feature extraction for vision-based approaches is computationally expensive [22].

In this study, images captured by webcam have been utilized. The purpose of utilizing a webcam is that it is comparatively simple for anyone to get one, and the price is affordable. The fact that one does not require expensive equipment, such as the leap motion sensor or 3D camera stated earlier is undoubtedly an essential benefit of using a webcam system. For the feature extraction, a recently developed coordinate estimator called media-pipe [25], has been utilized in this research to obtain 21 coordinate estimators of hands from 2D images. After trying several coordinate estimators this one was chosen because it is relatively less prone to collapse and can estimate coordinates cleanly, making it suitable for this research. From the coordinate estimators or joint points, distance-based and angle-based features have been extracted. After that, the support vector machine and the light gradient boosting machine have been used for recognition. Because of using distance-based and angle-based features the feature extraction process is not computationally expensive like convolutional neural networks or color-based mechanisms. Moreover, the feature extraction procedure is able to extract 3D skeletal joint points from a 2D image without using a leap motion controller, 3D camera, or any other specialized devices or sensors.

That means our proposed system is ensuring the strengths of both sensor-based and vision-based approaches such as free movement, high accuracy, skeletal points extraction from 2D images, and contactless and affordable recognition. At the same time, our proposed system is omitting the weaknesses of both sensor-based and vision-based approaches, such as usage of expensive devices, usage of costly cameras, high computational complexity, and lower accuracy. Since this study uses a webcam, similar studies that classify characters from RGB cameras or images will be used for comparison. One of the studies has a very high result of 99.31% for Massey dataset [26], and this result is one of the indicators.

Similar to natural languages, sign language also holds specific grammar and vocabulary [27]. However, despite having similarities and notable connections, sign languages all over the world are not widely the same and not mutually recognized [27]. Depending on the community, the corresponding sign language also differs in terms of gestures. In this research, American sign language has been considered as it is utilized by the American and Canadian deaf community consisting of approximately 250,000 to 500,000 Americans and some Canadians [28].

## 2. Literature Review

In this section, related works will be discussed considering both sensor-based and vision-based approaches. Researches on hand tracking and hand pose recognition have also been discussed here as sign language recognition is an application of hand pose recognition.

One recent study suggested a novel approach for textual input in which the authors conducted an air-writing recognition using smart bands [29]. In [29], the authors proposed a user-dependent method based on k-nearest neighbors (KNN) with dynamic tree wrapping (DTW) as the distance measure and a user-independent method based on a convolutional neural network (CNN) that achieved 89.2% and 83.2% average accuracy, respectively. Apart from the smart bands, Kinect sensors have been being utilized by researchers for a long time now. Earlier research suggested that up to 98.9% average recognition rate can be achieved by capturing the letters with Kinect sensors and recognizing them by using dynamic programming (DP) matching based on inter-stroke information [30]. However, mastering the technique of writing in the air and the usage of Kinect sensors requires specialized training, experience, and a suitable environment with the necessary equipment. Another earlier research suggested a similar approach where the authors captured the alphanumeric characters written in the air through a video camera instead of Kinect sensors and further experimentation by dynamic programming matching revealed an overall accuracy of 75% [31]. The main limitation of this study was the determination of starting and ending points for input and extraction of the user’s hand region in each picture frame.

A more recent study proposed an RGB and RGB-D static gesture recognition mechanism that utilized a fine-tuned VGG19 model after capturing the gestures with Kinect sensors and reported a recognition rate of 94.8% [32]. Based on the successful recognition of hand gestures, soon the techniques of recognizing hand gestures have been adapted for sign language alphabet recognition. Microsoft Kinect has been utilized for American sign language recognition where the authors proposed a random forest classifier on segmented hand configuration and obtained 90% accuracy [33]. A recent study utilized InceptionV3, a convolutional neural network model, to obtain 90% validation accuracy on the American sign language dataset containing 24 characters from the American sign alphabet [34]. One of the recent works on American sign language recognition proposed a restricted Boltzmann machine (RBM) fusing mechanism and reported 99.31%, 97.56%, 90.01%, and 98.13% recognition accuracy for Massey dataset, ASL finger spelling A dataset, NYU dataset, and ASL finger spelling dataset of the Surrey University, respectively [26].

Another popular way of hand gesture recognition is via leap motion. Recent work on British sign language recognition suggested a multimodality approach by fusing two artificial neural networks (ANN) and 94.44% overall accuracy was reported by utilizing the leap motion [35]. Leap motion has also been utilized recently for the recognition of American sign language gestures in a virtual reality environment and the authors reported a mean accuracy of 86.1% [36]. In [36], the authors have utilized the data from the leap motion device and hidden Markov classifier (HMC) was utilized for the recognition process. In another work, the authors used a leap motion controller and convolutional neural network to achieve 80.1% accuracy [37]. Moreover, the leap motion controller with support vector machine (SVM) and the deep neural network (DNN) has been applied on 36 American signs beforehand with a reported accuracy of 72.79% and 88.79%, respectively [38].

Apart from the above-mentioned approaches, some other schemes for the recognition of American sign language have been proposed beforehand. A work on static American signs utilized a skin-color modeling technique and convolutional neural network to achieve 93.67% accuracy [39]. Another research utilized a deep neural network on RGB images with a squeezenet architecture to make it suitable for mobile devices and achieved an overall accuracy of 83.28% [40]. Skeletal data and distance descriptors with TreeBag and neural network (NN) classifiers have been utilized to achieve 90.7% accuracy [41]. Another work proposed a recognition system for the sign language alphabet that utilizes geometrical features with an artificial neural network and achieved 96.78% accuracy [42]. Besides, neuromorphic sensors with the artificial neural network have previously reported 79.58% accuracy for 24 American signs [43]. Furthermore, a convolutional neural network with multiview augmentation and inference fusion has been used to achieve 93% accuracy [44]. Table 1 presents the related works with their corresponding approach, classifiers, and recognition rate for a better understanding. It can be observed that the sensor-based approaches have achieved higher accuracy although they are costly. Additionally, some vision-based approaches have utilized CNNs to achieve relatively higher accuracy. However, in such cases, the computational complexity has increased exponentially as well.

## 3. Materials and Methods

In this section, first, the details of the dataset have been discussed. After that, the details about the hand pose estimation, distance, and angle-based features, and two classification methods (SVM and light GBM) have been described.

### 3.1. Dataset Description

**Figure 1:**
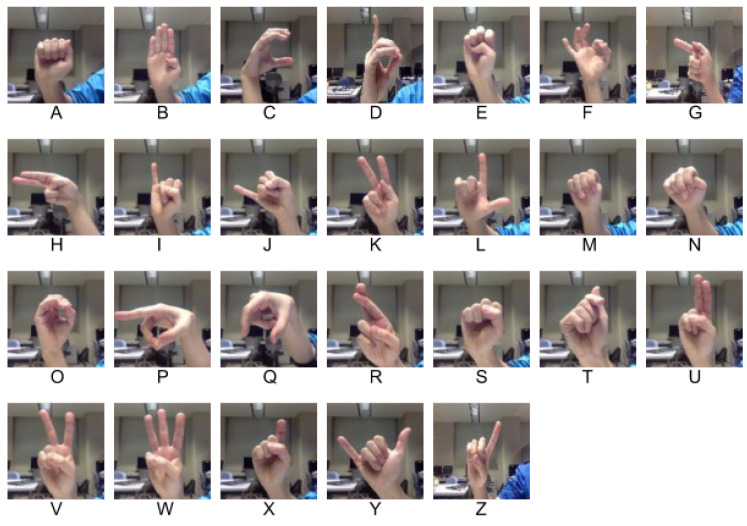
26 characters of the American sign language alphabet.

**Figure 2:**
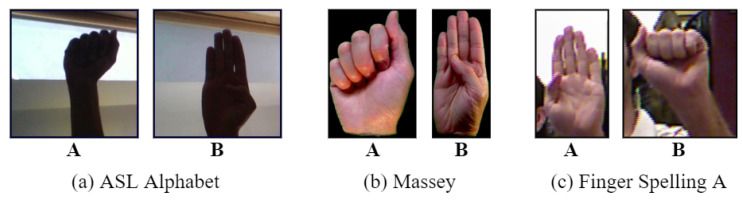
American sign language sample images for A and B from ASL alphabet (**a**), Massey (**b**) and finger spelling A (**c**) datasets.

American sign language, popularly known as ASL [45] is a sign language used in English-speaking countries, such as the United States and Canada, and it consists of 26 letters of the alphabet from A to Z that can be expressed with one hand and has been illustrated in Figure 1. In this study, a total of three datasets have been utilized. First, the ASL alphabet dataset from Kaggle [46] has been used for character recognition to evaluate the performance of more difficult data. The Massey dataset [47] has been utilized to compare the obtained results with the previous studies, which has produced the best recognition rate. In addition, the finger spelling A dataset [48] has been used in this study. Figure 2 shows similar samples from all three datasets for a better understanding of the similarity and complexity of the three considered datasets.

#### 3.1.1. ASL Alphabet Dataset

The first dataset used in this study is the ASL data [46], which contains the letters A to Z. In Figure 2a, it can be observed that the ASL alphabet dataset contains images that are difficult to distinguish, making it a very difficult dataset. Later, the experimental analysis will show that the proposed methodology works decently even for this difficult dataset. There are a total of 780,000 images in the dataset containing 3000 samples per class.

#### 3.1.2. Massey Dataset

Previously, researchers focused on the Massey dataset [47] to report the classification accuracy. Hence, in this study, the Massey dataset has been considered also for a fair comparison with previous work. The dataset contains all 26 letters of the American sign alphabet. However, it is relatively easy to perform sign alphabet recognition on the Massey dataset as the areas other than the hand in the images are black and the hand condition is shown clearly. The dataset contains a total of 1815 images. Apart from 65 samples of the class T, all the other 25 classes have 70 samples each.

#### 3.1.3. Finger Spelling A Dataset

The finger spelling A [48] dataset is another popular dataset that has been considered in this study. From Figure 2c, it can be observed from the figure that the dataset is characterized by a less clear image quality of the hand than the Massey dataset. In addition, this dataset has both RGB and depth images. However, only the RGB images have been used in this study. There are a total of 24 characters in this dataset. The authors of the dataset decided not to include J and Z as they are motion-based signs and the study was about static signs. There are a total of 65,774 images. The number of images per class varies from 2615 to 3108.

### 3.2. Feature Extraction

Feature extraction has been used to recognize the ASL alphabet in this study. The number of obtained coordinates of the joints is 21 in 3D space containing values of X, Y, and Z-axis, and these coordinates have been utilized to extract new features. This is because there may arise some problems if the coordinates are left as they are. For example, if the hand is on the right edge of the camera or image, the output will be presented as a different value even if it has the same signature as the hand on the left edge. Therefore, we need features that are not affected by the location on the screen. In addition, there are some signs in the American sign language that have the same hand shape but represent different characters depending on the degree of tilt, therefore, it is needed to extract features that work effectively even in those cases. In this study, both the distance-based features and the angle-based features were extracted from the initial joint points which has been described in the later sections.

#### 3.2.1. Hand Pose Estimation

**Figure 3:**
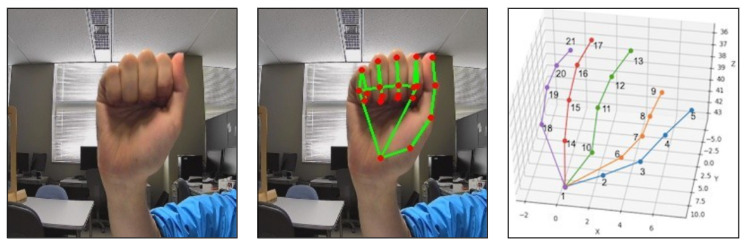
Using media-pipe API to obtain 21 joint points. The image in the left is an input image, the image in the middle is presenting the estimated joints and the image in the right is showing the joint order.

Media-pipe hands is an API developed by Google to estimate the coordinates of each joint from a web camera [25]. It can also estimate the coordinates of joints from RGB images. The output produced by the API consists of 21 points, each with 3D (XYZ) coordinates. The order of the coordinates is as follows: the first coordinate is for the wrist which is the bottom point, from there the thumbs coordinates are in the order 1–5 from the bottom, then the index fingers are in the order 6–9 from the bottom point 1, and so on. The position of the wrist and other joints is not fixed, and the coordinates of each joint point change as they move with the movement of the hand. Figure 3 illustrates a sample input image, the estimated joint points and the order of the joint points.

#### 3.2.2. Distance-Based Features

In order to extract features that are not affected by the screen position, first, the distances between the number of 21 coordinates are calculated. However, the distances between neighboring joints were not considered. The distance between two joint points *i* and *j* can be obtained by using Equation (1).

(1) $$d_{ij}=\sqrt{{\left(x_{i}-x_{j}\right)}^{2}+{\left(y_{i}-y_{j}\right)}^{2}+{\left(z_{i}-z_{j}\right)}^{2}}$$

**Figure 4:**
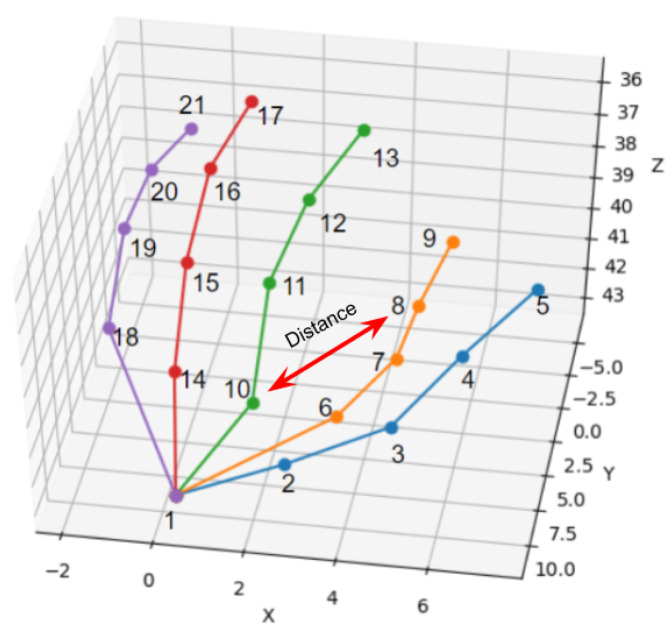
An example of distance-based features. Here, the distance between 8th and 10th joint points is being measured.

Figure 4 illustrates the distance between the 8th and 10th joint points. Here, neighboring joints are the joints that are connected by bones. For example, in the case of the third joint, the second and fourth joints would be the adjacent joints. Since the relative positions of neighboring joints are always fixed by the bones, the distances between adjacent joints do not change even if the formation of the hand varies. Hence, the distances between adjacent joints will not have any impact on the classification as they will produce the same distance value every time regardless of the hand position or formation in the image. If neighboring joints are excluded, 190 features can be obtained from each image. Table 2 presents all possible 190 features and how they are obtained. It can be noticed that for points 20 and 21 joint points, the sets are empty. This is because the expected pairs to be formed considering 20 and 21 joint points as the starting point has already been covered by the previous pairs.

Although while using the distance between joints, the problem with the location is solved, the problem with the size of the object is still there. This is because if the recognized object is large, the distance between each joint will be large, and if the object is small, the distance between each joint will be small. Therefore, normalization of the obtained distance values was performed to solve this problem.

To normalize the data, z-score normalization has been utilized [49], which converts the mean of the original data to 0 and the standard deviation to 1. Max–min normalization was not chosen as it sets the maximum value to 1 and minimum value to 0 by not changing the overall ratio of the values. That means, for a larger hand in the image the max–min normalized distances will still be larger than a smaller hand scenario. However, z-score normalization is capable of tackling such dilemmas. Hence, z-normalization was decided upon for use. For the data in consideration, z-normalization can be performed with the assistance of Equation (2).

(2) $$z-normalization=\frac{data-{data}_{mean}}{{data}_{std}}$$

#### 3.2.3. Angle-Based Features

The feature values of how much the hand is tilted were calculated as the angle-based features. The direction vectors between the coordinates of each joint were calculated, as well as how much each vector was tilted from the X, Y, and Z-axis directions. Figure 4 illustrates this process where a vector has been created by connecting the 6th and 11th joint points. After that, the angles between the vector and the coordinates ($\vec{x}$, $\vec{y}$, and $\vec{z}$ vectors) have been calculated. Since the number of joints to be estimated is 21, a total of 210 vectors can be created, and three angle-based features can be calculated for each vector, resulting in a total of 630 angle-based features. Table 3 illustrates all possible 210 scenarios and the extraction of 630 angle-based features. Similarly to distance-based features, it can be noticed that for the joint point number 21 the set is empty. This is because the expected pairs have already been covered up by the earlier joint points.

These features are useful and the classifier is expected to have an advantage while the recognition process when the signs that have the same shape but different tilts based on the inclination of the hand is under consideration. For example, in this study, I and J are two such classes and the angle-based features are useful for such letters. While considering the distance-based features, both letters will produce the same distance-based features as apart from the tilt, the shape is the same. As a result, the distances between the joints do not change and the classifier will not be able to find a difference based on the distance-based features. However, the angle-based features can eliminate this problem. Hence, the angle from the axis is expected to be important.

**Figure 5:**
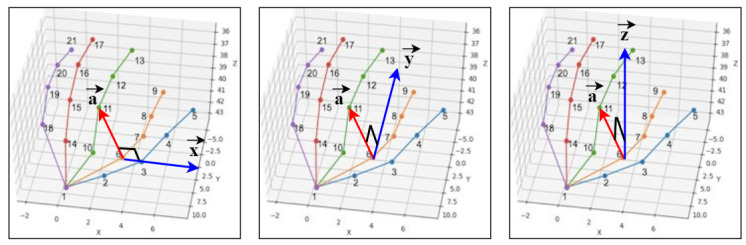
Calculation of angle-based features. Here, $\vec{a}$ is a vector created by 6th and 11th joints. After that, the angles between $\vec{a}$ and X-axis (**left**), Y-axis (**middle**), and Z-axis (**right**) have been calculated.

Additionally, since the angle information is not affected by the size of the hand, the extracted features do not require normalization as compared to the distance-based features described beforehand, and, as a result, the effect of the size of the hand will be reduced. The calculation method is to first calculate the direction vector between two points. The angle between the vectors can be calculated using the direction vector and the vectors in the X, Y, and Z-axis directions. Figure 5 illustrates extraction of such angles. The calculation method used was to calculate the cosine of the angle between the two spatial vectors. Suppose, we have two vectors $\vec{a}=\left(a_{1},a_{2},a_{3}\right)$ and $\vec{b}=\left(b_{1},b_{2},b_{3}\right)$. The angle between these two spatial vectors can be calculated by using Equation (3).

(3) $$cos\theta_{ab}=\frac{a_{1}b_{1}+a_{2}b_{2}+a_{3}b_{3}}{\sqrt{{a_{1}}^{2}+{a_{2}}^{2}+{a_{3}}^{2}}\sqrt{{b_{1}}^{2}+{b_{2}}^{2}+{b_{3}}^{2}}}$$

The method of calculating the inclination from the X-axis is to calculate vector $\vec{b}$ as vector (1,0,0) in the X-axis direction, which is expressed in Equation (4). Similarly, the method of calculating the inclinations from the Y-axis and X-axis is to calculate vector $\vec{b}$ as vector (0,1,0) and (0,0,1) in the Y-axis and Z-axis directions, which is expressed in Equations (5) and (6), respectively.

(4) $$cos\theta_{x}=\frac{a_{1}}{\sqrt{{a_{1}}^{2}+{a_{2}}^{2}+{a_{3}}^{2}}}$$

(5) $$cos\theta_{y}=\frac{a_{2}}{\sqrt{{a_{1}}^{2}+{a_{2}}^{2}+{a_{3}}^{2}}}$$

(6) $$cos\theta_{z}=\frac{a_{3}}{\sqrt{{a_{1}}^{2}+{a_{2}}^{2}+{a_{3}}^{2}}}$$

### 3.3. Classification

For classification, two methods, support vector machine (SVM) and light gradient boosting machine (GBM) have been utilized. SVM works well for unstructured and semi-structured high-dimensional datasets. With an appropriate Kernal function, SVM can solve complex problems. Unlike neural networks, SVM is not solved for local optima. SVM models have generalization in practice and, therefore, the risk of over-fitting is less in SVM. On the other hand, light GBM has faster training speed, lower memory usage, better performance than any other boosting algorithms, is compatible with large datasets, and supports parallel learning. Due to all these reasons, in this research, we chose both SVM and light GBM.

#### 3.3.1. Support Vector Machine

SVM is a pattern recognition model that utilizes supervised learning [50], and in this study, it has been utilized for classification. Support vector machine is a method to construct a pattern discriminator using linear input elements. From the training data, the parameters of the linear input elements are learned based on the criterion of finding the margin-maximizing hyperplane that maximizes the distance to each data point. The kernel used in this study is represented by Equation (7) where $X_{1}$ and $X_{2}$ are two points, *K* denotes kernel and $\left|\right|X_{1}-X_{2}\left|\right|$ denotes the Euclidian distance between the two points.

(7) $$K\left(X_{1},X_{2}\right)=e^{-\gamma {\left|\right|X_{1}-X_{2}\left|\right|}^{2}}$$

The support vector machine has parameters and in order to optimize the parameters, parameter tuning was performed. Grid search has been used to find the optimal values of cost (C) and gamma parameters in this research.

#### 3.3.2. Light Gradient Boosting Machine

**Figure 6:**
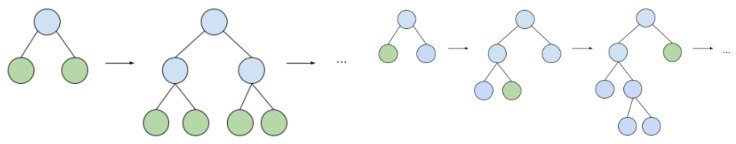
Two types of training: level-wise training (**left**), leaf-wise training (**right**).

Light GBM is a machine learning framework for gradient boosting based on the decision tree algorithm [51]. Gradient boosting is an ensemble learning method that combines multiple weak learners (in the case of light GBM, decision trees) into one, using ’boosting’. Before the arrival of light GBM, gradient boosting, called XGboost, was the mainstream method. Normal decision tree models, including Xgboost, are trained hierarchically. Light GBM uses leaf-wise learning, which is more efficient because it does not require unnecessary learning. Therefore, light GBM solves the drawback of gradient boosting such as XGboost, which has high prediction accuracy but a long computation time. Figure 6 illustrates both level-wise learning and leaf-wise learning.

### 3.4. Experimental Settings and Evaluation Metric

Each of the three datasets were divided into train set and test set, having 20% data in the test set. While tuning the support vector machine and light GBM, 5-fold cross validation has been utilized. Accuracy has been used as the evaluation metric in this research which is denoted by,

(8) $$Accuracy=\frac{TP+TN}{TP+TN+FP+FN}$$

Here, TP = True positive, FP = False positive, TN = True negative, and FN = False negative.

## 4. Experimental Analysis

This section starts with the details on parameter tuning. After that, experimental settings, evaluation metric and result analysis have been presented, along with a comparison with previous works. Later, the necessity of both the distance-based, and angle-based features on the overall performance has been discussed.

### 4.1. Parameter Turning

In this study, SVM and light GBM has been used to classify the ASL alphabet. To obtain the best parameters, we used grid search to select the parameters. The parameters searched were cost (C) values and Gamma values for SVM. Table 4 shows the parameters search space for SVM classifier. Table 5 shows the selected C and Gamma values after performing parameter tuning for each of the datasets. Grid search was also applied for selecting the best parameters for light GBM as well. The parameters searched were the number of leaves, learning rate, minimum child samples, and the number of estimators. Table 6 presents the parameters search space and Table 7 presents the selected parameters for each of the datasets while using the light GBM.

### 4.2. Results Analysis

In this study, two types of classifiers, SVM and light GBM have been used. SVM has been used as the main classifier, while light GBM has been utilized for comparison. There are two types of features, distance-based features, and angle-based features. Results after applying SVM and light GBM are illustrated in Table 8. From Table 8, it can be observed that when used alone, the angle-based features gave better results. In ASL, there are letters that have the same shape but different inclinations to express different characters. The distance-based features may be able to determine the shape but not the inclination. Therefore, the performance increased when angle-based features are used that can also determine the degree of inclination. Next, from Table 8 it can also be observed that the results are better when both distance-based and angle-based features are used than when used individually. Although the shape of the hand can be imagined from the inclination, it is still possible to estimate the shape of the hand more clearly with the distance features. Therefore, combining the two features led to further improvement in the accuracy which can be observed in Table 8. In addition, Table 9 presents average hand pose estimation time, average feature extraction time, prediction time per sample, recognized frames per second, and required memory to load final trained model for all three datasets using SVM while considering both distance-based and angle-based features. Here, all times are measured in seconds. It can be seen that the proposed system can recognize at least 62 samples per second which indicates that the proposed system is suitable for real-time gesture recognition. A Kaggle CPU environment, i.e., 2 CPU cores, 16 Gigabytes of RAM, 20 Gigabytes of disk space, was utilized while all experimentation.

### 4.3. Comparison with Previous Studies

In this section, the comparison between this study and previous studies will be discussed. There are two datasets that we are comparing in this study, one is the Massey dataset and the other one is the finger spelling A dataset as these two datasets have been used in previous studies. As can be seen from Table 10, the two datasets in this study showed better results than the previous studies. Specifically, the obtained accuracy of the Massey dataset is 99.39% and the accuracy of the finger spelling A dataset is 98.45%, which is higher than the previous studies. Then, 87.60% accuracy was achieved on the ASL alphabet dataset, which is considered to be a difficult dataset to classify. By using the coordinate estimation method used in this study, it is possible to obtain 3D information that cannot be obtained from 2D images, and this 3D information is important because it allows to easily identify important features regarding joint points that are difficult to identify in 2D. For example, if a person is grasping his or her hand, 3D information is much more useful in identifying the hand because it holds the information more clearly.

There are cameras that can obtain 3D information, such as leap motion and cameras equipped with depth sensors. However, in this research, we used images captured by a webcam, a camera that does not provide three-dimensional information like a depth camera. The reason why we considered webcam inputs is that it has the advantage of being easier to use than the above-mentioned cameras. Moreover, leap motion and depth sensors are more expensive. Web cameras, on the other hand, are inexpensive, and since even laptops are equipped with them, it is not difficult to get and use one. Therefore, we believe that obtaining good recognition rates even with a web camera can be highly beneficial for ASL recognition which will have a great impact on future research.

### 4.4. Necessity of Distance-Based Features, Angle-Based Features, and Both

**Figure 7:**
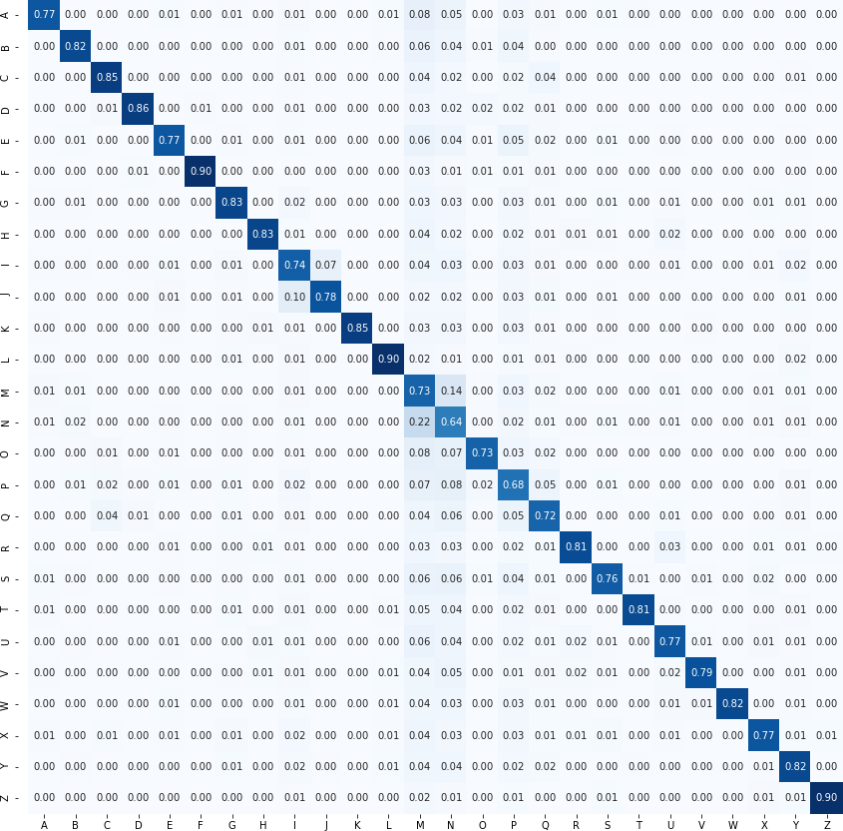
Confusion matrix for distance-based features.

**Figure 8:**
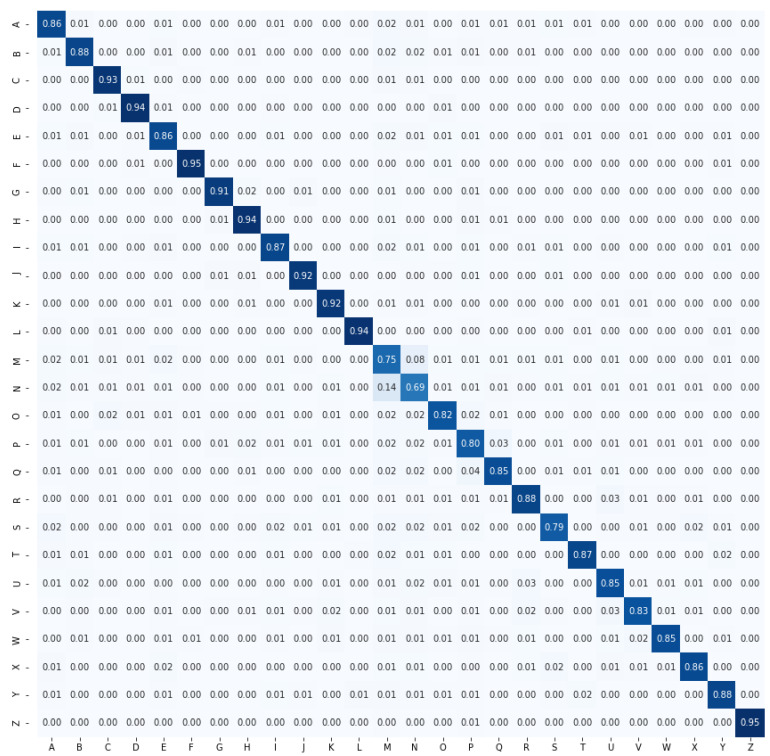
Confusion matrix for angle-based features.

In this study, two types of features are used for character recognition. One is the features using the distance between joints, and the other one is angles. Distance-based features can imagine the shape of the hand more clearly whereas angle-based features can imagine the tilt along with the shape. Normally, the recognition rate would be higher for the distance-based features if the classes of ASL solely depended on the shape of the hand. However, in ASL, some signs have the same shape but different inclinations to represent different letters. For example, the I and J have the same shape but different inclinations. Hence, specifically for ASL recognition, the angle-based features achieved better performance than the distance-based features. Figure 7 and Figure 8 illustrate the confusion matrices for distance-based features and angle-based features, respectively. A clear difference in performance can be seen here. Specifically, I and J also showed a difference in character recognition accuracy. When only distance-based features were used, the recognition rate of I was 74% and that of J was 78%. However, when angle information was used, the recognition rate of I improved to 87% and that of J to 92%. This phenomenon indicates the importance of the inclusion of angle-based features.

However, it is also true that distance-based features can imagine the hand shape more clearly than the angle-based features. As in ASL, many letters have different hand shapes, distance-based features can often be highly useful. For example, sometimes the hand may tilt to some degrees on the left or right unintentionally where tilting is not necessary. For those cases, the angle-based features can face problems in classification. However, this problem can be tackled by distance-based features. Therefore, the better choice seemed to combine both the distance-based and angle-based features. It turned out that combining both features indeed can boost the performance which has been reported beforehand. Table 8 illustrates the difference between using these features individually and in combination.

## 5. Conclusions

In this study, we used images obtained from a web camera to recognize sign characters in ASL. However, instead of just using the images, we estimated the coordinates of the hand joints from the images and used the estimated coordinates for recognition. Then, features were generated from the estimated coordinates, and character recognition was performed based on these features. The features we created were based on the distance between the joints and the angle between the direction between the two joints and the X, Y, and Z-axis. By using these features, it was expected that the complex shape of the hand could be easily represented and that the results would be better than using the images themselves. The results were as expected, and the method used in this study performed very well for sign language recognition in ASL. The experiments also showed that the accuracy of our method was better than that of previous papers. We believe this will be a great contribution to the field of character recognition. So far, there has been a great demand for systems that can input text without touching things, as is currently being researched for contactless text input systems. We hope this research will have a great impact on this field and will contribute to it. Another difference between this research and existing research is that, as mentioned earlier, this research considered webcam inputs which is not expensive and easier to get. In the future, we are thinking of recognizing not only ASL but also sign characters from other languages. In addition, the system used in this study can be applied not only to sign language recognition but also to air writing, which is the recognition of characters by writing them in the air. This indicates the diverse applications of this study and the potential to contribute greatly to future researches.

## Figures and Tables

**Table 1:** Previous works performances at a glance including the approach and classifier used.

Reference	Approach	Classifier	Recognition Rate
[29]	Smart Bands ${}^{1}$	KNN with DTW	89.20%
[29]	Smart Bands ${}^{1}$	CNN	83.20%
[30]	Kinect ${}^{1}$	DP Matching	98.90%
[31]	Video Camera ${}^{2}$	DP Matching	75.00%
[32]	Kinect ${}^{1}$	VGG19	94.80%
[33]	Kinect ${}^{1}$	Random Forest	90.00%
[34]	Direct Images ${}^{2}$	InceptionV3	90.00%
[26]	Images from Massey Dataset ${}^{2}$	RBM	99.31%
[26]	Images from Finger Spelling A Dataset ${}^{2}$	RBM	97.56%
[26]	Images from NYU Dataset ${}^{2}$	RBM	90.01%
[26]	Images from ASL Fingerspelling Dataset of Surrey University ${}^{2}$	RBM	98.13%
[35]	Leap Motion Camera ${}^{1}$	ANN	94.44%
[36]	Leap Motion in Virtual Reality Environment ${}^{1}$	HMC	86.10%
[37]	Leap Motion Controller ${}^{1}$	CNN	80.10%
[38]	Leap Motion Controller ${}^{1}$	SVM	72.79%
[38]	Leap Motion Controller ${}^{1}$	DNN	88.79%
[39]	Skin Color Modeling ${}^{2}$	CNN	93.67%
[40]	Direct Images ${}^{2}$	DNN with Squeezenet	83.28%
[41]	Skeletal Data and Distance Descriptor ${}^{1}$	TreeBag & NN	90.70%
[42]	Geometrical Features ${}^{1}$	ANN	96.78%
[43]	Neuromorphic Sensor ${}^{1}$	ANN	79.58%
[44]	Multiview Augmentation & Inference Fusion ${}^{1}$	CNN	93.00%

^1^ Followed sensor-based approaches; ^2^ Followed vision-based approaches.

**Table 2:** Considered set of starting and ending joint points for calculating distance-based features.

Starting Joint Number	Set of Two Joint Numbers for Measuring Distance by Considering Starting Joint Number as a First Joint Number	Number of Distance-Based Features
1	{(1,3), (1,4), (1,6), (1,7), (1,8), (1,10), (1,11), (1,12), (1,14), (1,15), (1,16), (1,18), (1,19), (1,20), (1,21)}	15
2	{(2,4), (2,5), (2,6), (2,7), (2,8), (2,9), (2,10), (2,11), (2,12), (2,13), (2,14), (2,15), (2,16), (2,17), (2,18), (2,19), (2,20), (2,21)}	18
3	{(3,5), (3,6), (3,7), (3,8), (3,9), (3,10), (3,11), (3,12), (3,13), (3,14), (3,15), (3,16), (3,17), (3,18), (3,19), (3,20), (3,21)}	17
4	{(4,6), (4,7), (4,8), (4,9), (4,10), (4,11), (4,12), (4,13), (4,14), (4,15), (4,16), (4,17), (4,18), (4,19), (4,20), (4,21)}	16
5	{(5,6), (5,7), (5,8), (5,9), (5,10), (5,11), (5,12), (5,13), (5,14), (5,15), (5,16), (5,17), (5,18), (5,19), (5,20), (5,21)}	16
6	{(6,8), (6,9), (6,10), (6,11), (6,12), (6,13), (6,14), (6,15), (6,16), (6,17), (6,18), (6,19), (6,20), (6,21)}	14
7	{(7,9), (7,10), (7,11), (7,12), (7,13), (7,14), (7,15), (7,16), (7,17), (7,18), (7,19), (7,20), (7,21)}	13
8	{(8,10), (8,11), (8,12), (8,13), (8,14), (8,15), (8,16), (8,17), (8,18), (8,19), (8,20), (8,21)}	12
9	{(9,10), (9,11), (9,12), (9,13), (9,14), (9,15), (9,16), (9,17), (9,18), (9,19), (9,20), (9,21)}	12
10	{(10,12), (10,13), (10,14), (10,15), (10,16), (10,17), (10,18), (10,19), (10,20), (10,21)}	10
11	{(11,13), (11,14), (11,15), (11,16), (11,17), (11,18), (11,19), (11,20), (11,21)}	9
12	{(12,14), (12,15), (12,16), (12,17), (12,18), (12,19), (12,20), (12,21)}	8
13	{(13,14), (13,15), (13,16), (13,17), (13,18), (13,19), (13,20), (13,21)}	8
14	{(14,16), (14,17), (14,18), (14,19), (14,20), (14,21)}	6
15	{(15,17), (15,18), (15,19), (15,20), (15,21)}	5
16	{(16,18), (16,19), (16,20), (16,21)}	4
17	{(17,18), (17,19), (17,20), (17,21)}	4
18	{(18,20), (18,21)}	2
19	{(19,21)}	1
20	{}	0
21	{}	0

**Table 3:** Set of vectors obtained when each joint point is considered as a starting point for measuring angle-based features. Here, $p_{i}$ means joint number *i*.

Starting Joint Number	Set of Vectors Formed by Taking Starting Joint Number as First Point and Other Joints as Second Points	Number of Angle-Based Features
1	{$\vec{p_{1}p_{2}}$, $\vec{p_{1}p_{3}}$, $\vec{p_{1}p_{4}}$, $\vec{p_{1}p_{5}}$, $\vec{p_{1}p_{6}}$, $\vec{p_{1}p_{7}}$, $\vec{p_{1}p_{8}}$, $\vec{p_{1}p_{9}}$, $\vec{p_{1}p_{10}}$, $\vec{p_{1}p_{11}}$, $\vec{p_{1}p_{12}}$	20×3=60
	$\vec{p_{1}p_{13}}$, $\vec{p_{1}p_{14}}$, $\vec{p_{1}p_{15}}$, $\vec{p_{1}p_{16}}$, $\vec{p_{1}p_{17}}$, $\vec{p_{1}p_{18}}$, $\vec{p_{1}p_{19}}$, $\vec{p_{1}p_{20}}$, $\vec{p_{1}p_{21}}$}	
2	{$\vec{p_{2}p_{3}}$, $\vec{p_{2}p_{4}}$, $\vec{p_{2}p_{5}}$, $\vec{p_{2}p_{6}}$, $\vec{p_{2}p_{7}}$, $\vec{p_{2}p_{8}}$, $\vec{p_{2}p_{9}}$, $\vec{p_{2}p_{10}}$, $\vec{p_{2}p_{11}}$, $\vec{p_{2}p_{12}}$	19×3=57
	$\vec{p_{2}p_{13}}$, $\vec{p_{2}p_{14}}$, $\vec{p_{2}p_{15}}$, $\vec{p_{2}p_{16}}$, $\vec{p_{2}p_{17}}$, $\vec{p_{2}p_{18}}$, $\vec{p_{2}p_{19}}$, $\vec{p_{2}p_{20}}$, $\vec{p_{2}p_{21}}$}	
3	{$\vec{p_{3}p_{4}}$, $\vec{p_{3}p_{5}}$, $\vec{p_{3}p_{6}}$, $\vec{p_{3}p_{7}}$, $\vec{p_{3}p_{8}}$, $\vec{p_{3}p_{9}}$, $\vec{p_{3}p_{10}}$, $\vec{p_{3}p_{11}}$, $\vec{p_{3}p_{12}}$, $\vec{p_{3}p_{13}}$	18×3=54
	$\vec{p_{3}p_{14}}$, $\vec{p_{3}p_{15}}$, $\vec{p_{3}p_{16}}$, $\vec{p_{3}p_{17}}$, $\vec{p_{3}p_{18}}$, $\vec{p_{3}p_{19}}$, $\vec{p_{3}p_{20}}$, $\vec{p_{3}p_{21}}$}	
4	{$\vec{p_{4}p_{5}}$, $\vec{p_{4}p_{6}}$, $\vec{p_{4}p_{7}}$, $\vec{p_{4}p_{8}}$, $\vec{p_{4}p_{9}}$, $\vec{p_{4}p_{10}}$, $\vec{p_{4}p_{11}}$, $\vec{p_{4}p_{12}}$, $\vec{p_{4}p_{13}}$	17×3=51
	$\vec{p_{4}p_{14}}$, $\vec{p_{4}p_{15}}$, $\vec{p_{4}p_{16}}$, $\vec{p_{4}p_{17}}$, $\vec{p_{4}p_{18}}$, $\vec{p_{4}p_{19}}$, $\vec{p_{4}p_{20}}$, $\vec{p_{4}p_{21}}$}	
5	{$\vec{p_{5}p_{6}}$, $\vec{p_{5}p_{7}}$, $\vec{p_{5}p_{8}}$, $\vec{p_{5}p_{9}}$, $\vec{p_{5}p_{10}}$, $\vec{p_{5}p_{11}}$, $\vec{p_{5}p_{12}}$, $\vec{p_{5}p_{13}}$, $\vec{p_{5}p_{14}}$	16×3=48
	$\vec{p_{5}p_{15}}$, $\vec{p_{5}p_{16}}$, $\vec{p_{5}p_{17}}$, $\vec{p_{5}p_{18}}$, $\vec{p_{5}p_{19}}$, $\vec{p_{5}p_{20}}$, $\vec{p_{5}p_{21}}$}	
6	{$\vec{p_{6}p_{7}}$, $\vec{p_{6}p_{8}}$, $\vec{p_{6}p_{9}}$, $\vec{p_{6}p_{10}}$, $\vec{p_{6}p_{11}}$, $\vec{p_{6}p_{12}}$, $\vec{p_{6}p_{13}}$, $\vec{p_{6}p_{14}}$	15×3=45
	$\vec{p_{6}p_{15}}$, $\vec{p_{6}p_{16}}$, $\vec{p_{6}p_{17}}$, $\vec{p_{6}p_{18}}$, $\vec{p_{6}p_{19}}$, $\vec{p_{6}p_{20}}$, $\vec{p_{6}p_{21}}$}	
7	{$\vec{p_{7}p_{8}}$, $\vec{p_{7}p_{9}}$, $\vec{p_{7}p_{10}}$, $\vec{p_{7}p_{11}}$, $\vec{p_{7}p_{12}}$, $\vec{p_{7}p_{13}}$, $\vec{p_{7}p_{14}}$, $\vec{p_{7}p_{15}}$	14×3=42
	$\vec{p_{7}p_{16}}$, $\vec{p_{7}p_{17}}$, $\vec{p_{7}p_{18}}$, $\vec{p_{7}p_{19}}$, $\vec{p_{7}p_{20}}$, $\vec{p_{7}p_{21}}$}	
8	{$\vec{p_{8}p_{9}}$, $\vec{p_{8}p_{10}}$, $\vec{p_{8}p_{11}}$, $\vec{p_{8}p_{12}}$, $\vec{p_{8}p_{13}}$, $\vec{p_{8}p_{14}}$, $\vec{p_{8}p_{15}}$	13×3=39
	$\vec{p_{8}p_{16}}$, $\vec{p_{8}p_{17}}$, $\vec{p_{8}p_{18}}$, $\vec{p_{8}p_{19}}$, $\vec{p_{8}p_{20}}$, $\vec{p_{8}p_{21}}$}	
9	{$\vec{p_{9}p_{10}}$, $\vec{p_{9}p_{11}}$, $\vec{p_{9}p_{12}}$, $\vec{p_{9}p_{13}}$, $\vec{p_{9}p_{14}}$, $\vec{p_{9}p_{15}}$	12×3=36
	$\vec{p_{9}p_{16}}$, $\vec{p_{9}p_{17}}$, $\vec{p_{9}p_{18}}$, $\vec{p_{9}p_{19}}$, $\vec{p_{9}p_{20}}$, $\vec{p_{9}p_{21}}$}	
10	{$\vec{p_{10}p_{11}}$, $\vec{p_{10}p_{12}}$, $\vec{p_{10}p_{13}}$, $\vec{p_{10}p_{14}}$, $\vec{p_{10}p_{15}}$, $\vec{p_{10}p_{16}}$	11×3=33
	$\vec{p_{10}p_{17}}$, $\vec{p_{10}p_{18}}$, $\vec{p_{10}p_{19}}$, $\vec{p_{10}p_{20}}$, $\vec{p_{10}p_{21}}$}	
11	{$\vec{p_{11}p_{12}}$, $\vec{p_{11}p_{13}}$, $\vec{p_{11}p_{14}}$, $\vec{p_{11}p_{15}}$, $\vec{p_{11}p_{16}}$	10×3=30
	$\vec{p_{11}p_{17}}$, $\vec{p_{11}p_{18}}$, $\vec{p_{11}p_{19}}$, $\vec{p_{11}p_{20}}$, $\vec{p_{11}p_{21}}$}	
12	{$\vec{p_{12}p_{13}}$, $\vec{p_{12}p_{14}}$, $\vec{p_{12}p_{15}}$, $\vec{p_{12}p_{16}}$, $\vec{p_{12}p_{17}}$	9×3=27
	$\vec{p_{12}p_{18}}$, $\vec{p_{12}p_{19}}$, $\vec{p_{12}p_{20}}$, $\vec{p_{12}p_{21}}$}	
13	{$\vec{p_{13}p_{14}}$, $\vec{p_{13}p_{15}}$, $\vec{p_{13}p_{16}}$, $\vec{p_{13}p_{17}}$, $\vec{p_{13}p_{18}}$, $\vec{p_{13}p_{19}}$, $\vec{p_{13}p_{20}}$, $\vec{p_{13}p_{21}}$}	8×3=24
14	{$\vec{p_{14}p_{15}}$, $\vec{p_{14}p_{16}}$, $\vec{p_{14}p_{17}}$, $\vec{p_{14}p_{18}}$, $\vec{p_{14}p_{19}}$, $\vec{p_{14}p_{20}}$, $\vec{p_{14}p_{21}}$}	7×3=21
15	{$\vec{p_{15}p_{16}}$, $\vec{p_{15}p_{17}}$, $\vec{p_{15}p_{18}}$, $\vec{p_{15}p_{19}}$, $\vec{p_{15}p_{20}}$, $\vec{p_{15}p_{21}}$}	6×3=18
16	{$\vec{p_{16}p_{17}}$, $\vec{p_{16}p_{18}}$, $\vec{p_{16}p_{19}}$, $\vec{p_{16}p_{20}}$, $\vec{p_{16}p_{21}}$}	5×3=15
17	{$\vec{p_{17}p_{18}}$, $\vec{p_{17}p_{19}}$, $\vec{p_{17}p_{20}}$, $\vec{p_{17}p_{21}}$}	4×3=12
18	{$\vec{p_{18}p_{19}}$, $\vec{p_{18}p_{20}}$, $\vec{p_{18}p_{21}}$}	3×3=9
19	{$\vec{p_{19}p_{20}}$, $\vec{p_{19}p_{21}}$}	2×3=6
20	{$\vec{p_{20}p_{21}}$}	1×3=3
21	{}	0

**Table 4:** Parameter search space for SVM classifier.

Parameter Name	Used Values for Grid Search
C	0.1, 1, 10, 100, 1000
gamma	scale *, 0.001, 0.0001

* ‘scale’ is the default parameter of gamma value implemented in sklearn’s SVM, which is automatically calculated from the number of training data and the variance of feature variables by using the formula 1/(number of features × variance of features) [52].

**Table 5:** Selected parameters of SVM for different datasets.

Dataset	Parameter	All Distance	All Angle	Both Distance and
		Features	Features	Angle Features
ASL Alphabet	C	1000	1000	1000
	gamma	0.001	0.001	scale
Massey	C	100	1000	1000
	gamma	0.01	scale	0.0001
Finger Spelling A	C	1000	1000	1000
	gamma	0.001	0.001	scale

**Table 6:** Parameter search space for light GBM classifier.

Parameter Name	Used Values for Grid Search
number of leaves	5, 10, 25, 50, 75, 100, 500, 1000
learning rate	0.1, 0.01, 0.001, 0.0001, 0.00001
minimum child samples	5, 10, 25, 50, 100, 500, 1000
number of estimators	10–250

**Table 7:** Selected parameters of light GBM for different datasets.

Dataset	Parameter	All Distance	All Angle	Both Distance and
		Features	Features	Angle Features
ASL Alphabet	No. of leaves	100	100	100
	learning rate	0.1	0.1	0.1
	min. child samples	25	25	25
	No. of estimators	12	13	14
Massey	No. of leaves	50	50	50
	learning rate	0.1	0.1	0.1
	min. child samples	25	25	25
	No. of estimators	86	82	200
Finger Spelling A	No. of leaves	100	100	100
	learning rate	0.1	0.1	0.1
	min. child samples	25	25	25
	No. of estimators	44	42	40

**Table 8:** Obtained experimental results for considered datasets by using SVM and light GBM.

Classifier	Dataset	All Distance	All Angle	Both Distance and
		Features (190)	Features (630)	Angle Features (820)
SVM	ASL Alphabet	81.20%	87.06%	**87.60%**
	Massey	98.56%	99.23%	**99.39%**
	Finger Spelling A	96.97%	97.63%	**98.45%**
Light GBM	ASL Alphabet	79.11%	86.01%	**86.12%**
	Massey	96.51%	97.25%	**97.80%**
	Finger Spelling A	94.50%	96.06%	**96.71%**

**Table 9:** Average hand pose estimation time (Average HPET), average feature extraction time (Average FET), prediction time per sample (PTPS), recognized frames per second (RFPS), and required memory to load final trained model (Req. Memory) for all three datasets using SVM. All times are measured in seconds.

Dataset	Samples	Avg. HPET	Avg. FET	PTPS	RFPS	Req. Memory
Massey	1815	0.011	0.003	0.014	71	4.04 MB
Finger Spelling A	65,774	0.01	0.002	0.015	66	47.97 MB
ASL Alphabet	780,000	0.011	0.002	0.016	62	115.14 MB

**Table 10:** Comparison with other existing works.

Dataset	Approach	Accuracy
Massey Dataset	CNN [53]	72.00%
	RBM [26]	99.31%
	**Proposed**	**99.39%**
Finger Spelling A Dataset	Random Forest [33]	90.00%
	InceptionV3 [34]	90.00%
	DNN with Squeezenet [40]	83.28%
	ANN [43]	79.58%
	CNN [44]	93.00%
	RBM [26]	98.13%
	**Proposed**	**98.45%**

## Data Availability

ASL Alphabet dataset is accessible at https://www.kaggle.com/grassknoted/asl-alphabet. Massey dataset can be found at https://www.massey.ac.nz/~albarcza/gesture_dataset2012.html. Finger Spelling A dataset is available at https://empslocal.ex.ac.uk/people/staff/np331/index.php?section=FingerSpellingDataset.
